# Neglect dyslexia: whole-word and within-word errors with parafoveal and foveal viewing

**DOI:** 10.1007/s00221-023-06708-4

**Published:** 2023-09-29

**Authors:** Timothy J. Rich, John Palmer

**Affiliations:** 1 Center for Stroke Rehabilitation Research, Kessler Foundation, 1199 Pleasant Valley Way, West Orange, NJ USA; 2 Department of Physical Medicine and Rehabilitation, Rutgers University, New Brunswick, NJ USA; 3 Department of Psychology, University of Washington, Seattle, WA USA

**Keywords:** Neglect dyslexia, Unilateral paralexias, Selective attention, Reading

## Abstract

Patients with left-sided neglect dyslexia often omit whole words positioned on the left, termed *whole-word errors*, or commit errors on the left-sided letters of words, termed *unilateral paralexias*. In addition, the errors have been shown to be exacerbated by simultaneously presented distractors, which has been interpreted as a failure of selective attention. In two experiments, we examined the dependency of these error types on parafoveal versus foveal viewing. The first experiment used a paradigm with parafoveal targets and distractors; the second a paradigm with foveal targets and parafoveal distractors. This enabled a separate evaluation of the influences of stimulus position within an egocentric frame, a two-word allocentric frame, and a within-word allocentric frame. First, regarding whole-word errors, we found the expected spatial and distractor effects with parafoveal targets and distractors. With foveal targets and parafoveal distractors, however, the spatial effect was effectively eliminated. Surprisingly, intrusions from the distractor word were common in distractor conditions. This is consistent with an egocentric account and not a two-word allocentric account. Second, we found that unilateral paralexias remained largely consistent regardless of spatial position or the presence of a distractor. Thus, there is a contrast in spatial and distractor effects between whole-word errors and unilateral paralexias. These results are consistent with three distinct deficits: an egocentric deficit across space resulting in whole-word errors, a failure of selective attention that results in whole-word intrusion errors, and a within-word allocentric deficit resulting in unilateral paralexias.

## Introduction

Up to 25% of right hemisphere stroke survivors experience left-sided neglect dyslexia (Lee et al. 2009; Pedrazzini and Ptak 2019; Esposito et al. 2021), the reading impairment experienced by some, but not all, patients with spatial neglect (Behrmann et al. 2002; Vallar et al. 2010). There are two primary types of errors in neglect dyslexia: *whole-word errors*, in which entire words positioned on the left side are omitted (Beschin et al. 2014); and *unilateral paralexias*, in which the initial (i.e., left-sided) letters of a word are substituted or omitted (Benson 1985; Ellis et al. 1987; Siéroff 2017).

The study of these error types has provided evidence for dissociable deficits of egocentric (i.e., viewer-centered) and allocentric (i.e., object-centered) spatial frames of reference (Ellis et al. 1987; Vallar et al. 2010; Ptak et al. 2016). Ptak et al. (2012) compared the frequency of whole-word errors to the frequency of unilateral paralexias during a single word reading task. Participants read aloud 40 words arranged in five staggered columns, giving the viewer the impression of a random arrangement, spanning the left-to-right margins of an A4 sheet of paper. Whole-word errors occurred on approximately 44% of words, which decreased incrementally from left to right. In contrast, unilateral paralexias occurred in approximately 9% of words but were evenly distributed across the page. Their findings are consistent with an explanation of two distinct spatial deficits underlying errors: one, a deficit of egocentric processing resulting in whole-word errors, and another, a deficit of allocentric processing within a word resulting in unilateral paralexias.

Further evidence supporting this dichotomy include manipulations of the egocentric frame of reference by rotating the head leftward relative to the stimulus, which resulted in improvements in whole-word errors but caused no such improvement in unilateral paralexias (Reinhart et al. 2010). Manipulations of the presentation of single words, such as vertically or mirror-reversed, have shown that, for some patients, unilateral paralexias occur in the early stages of word recognition (i.e., retinocentric frame of reference); for others in the later stages (i.e., stimulus- or word-centered frames of reference; Hillis and Caramazza 1995; see Haywood and Coltheart 2000 for a review).

However, spatial frames of reference are not used by the visual system in isolation. Rather, multiple egocentric and allocentric coordinate schemes overlap and interact to create a spatial representation of the environment and the objects within it (Behrmann and Tipper 1999; Halligan et al. 2003). To facilitate efficient and sequential reading of a magazine, for example, multiple allocentric frames are constructed for individual words, columns, and pages, which are all positioned within overlapping egocentric frames of the eyes, head, and trunk. Relatively few studies have explored the impact of the presentation of two words simultaneously on whole-word and unilateral paralexic errors, which is more representative of the challenges faced by individuals with neglect dyslexia during natural reading. Thus, much remains to be learned regarding the effects of distractor words on the accuracy of the recognition of a target word, as well as the influences of multiple, overlapping frames of reference.

In addition to the manipulation of the allocentric spatial characteristics of the stimulus, the presentation of two or more words introduces competition for selective attention between the words. Neglect dyslexic errors are modulated by these distractors. Many patients with unilateral brain damage who are able to report a contralesional target when presented in isolation are unable to detect or report a target in the same spatial location when presented alongside an ipsilesional distractor, a phenomenon known as *extinction*.

The traditional extinction paradigm involves the detection of stimuli in three conditions: two with a solitary unilateral stimulus presented on the left or the right side; and one with bilateral stimuli on the left and right side. Variations on this paradigm have shown that extinction is more likely for stimuli that are visually similar than dissimilar (Rafal et al. 2002; Ricci and Chatterjee 2004; Ptak and Schnider 2005) and for the task of stimulus identification than detection (Ricci and Chatterjee 2004).

Siéroff and Urbanski (2002) studied extinction in words using a variation of the traditional paradigm. They presented four-letter words either solitarily to the left or right of fixation, or simultaneously with a contralateral word. Patients showed effects of both space, with poorer accuracy for left-sided words than right-sided in all conditions; and distractors, with poorer accuracy for left-sided words in bilateral conditions. Their findings and those of similar studies with non-word stimuli (e.g., Geeraerts et al. 2005; Shalev et al. 2005), suggest that a distractor effect, i.e., a failure of selective attention, plays a role in neglect dyslexia when two or more words are presented simultaneously.

Complicating interpretations of extinction studies with words, there is a well-known right hemifield advantage for single word reading in neurotypical participants (Mishkin and Forgays 1952; Siéroff and Riva 2011). This asymmetry has been attributed to several different sources: the left hemisphere’s specialization for language (Bryden 1982; Hellige 1993; Behrmann and Plaut 2013), the eccentricity differences of the first, most informative letter of the word when presented in the right versus left visual hemifield (Kirsner and Schwartz 1986), or the habitual behaviors of readers of left-to-right scripts (Battista and Kalloniatis 2002).

A limitation of the traditional extinction paradigm is that it does not discern whether the distractor effect is due to the egocentric or allocentric position of the competing stimuli. Are left-sided words at a competitive disadvantage because of their spatial position relative to the viewer (i.e., their egocentric position) or relative to the right-sided word (i.e., their allocentric position within the two-word pair)? These two possibilities have been distinguished previously with non-word stimuli by Mattingley et al. (2000; Experiment 5), who reported a single patient who showed extinction for a left-sided peripheral target stimulus when flanked by a foveal distractor (thereby positioning the target on the relative left side of the stimulus pair) but no extinction for a central target flanked by a right-sided distractor. Their findings suggest that the distractor effects demonstrated by those with extinction are, at least in part, dependent on their position in contralesional egocentric space. However, the spatial contribution to a distractor effect for foveal words in patients with neglect dyslexia has yet to be explored.

A second limitation of the traditional extinction paradigm is that the parafoveal presentation of words renders the experimental task quite unlike natural reading, which largely depends on recognizing words in the fovea through serial fixations (Reichle et al. 2003). In addition, the visual system pre-processes upcoming words within a “moving window” that includes as many as 15 character spaces to the right of fixation (McConkie and Rayner 1975). Through a paradigm using gaze-contingent masking outside of the moving window, numerous studies have shown, for neurotypical readers, improved reading efficiency as the span of the moving window is increased (Rayner 2014). If, for those with neglect dyslexia, the accuracy of reading a foveal target word suffers due to the upcoming words on that line of text (i.e., on the relative right side of the target), the moving window—normally a facilitator of efficient reading—becomes, instead, a barrier.

### Overview of experiments

In this study, we used the traditional extinction paradigm and a modified paradigm to explore the effects of egocentric space, allocentric space, and distractors. In the modified paradigm, the target word was presented at the fovea with a parafoveal distractor to the left or right, to distinguish the effects of its position in egocentric space and its position within a two-word allocentric frame. If a distractor effect is dependent on the spatial position of the target word relative to the viewer, right-sided distractors would cease to induce errors with foveal targets. Conversely, if a distractor effect is dependent on the spatial position of the target word relative to the distractor, the effect from right-sided distractors would persist with foveal targets. We separately analyzed whole-word errors and unilateral paralexias to determine if the two error types share common spatial or distractor effects.

## General methods

### Participants

All participants provided informed consent prior to testing in accordance with the Declaration of Helsinki. Our experimental group was comprised of ten patients with right hemisphere stroke and left-sided neglect dyslexia. Six patients completed Experiment 1 and all ten completed Experiment 2. Patient demographic data are presented in Table 1. All patients were seen within a few weeks of stroke except for two (P-04 and P-10), who completed testing 85 weeks and 150 weeks post-stroke.

**Table 1:** Patient demographics

ID	Sex	Age (years)	Education (years)	Type of stroke	Location of stroke	Weeks post-stroke
P-01	M	48	10	R MCA aneurysm rupture	Frontal/temporal	5
P-04	F	69	16	R MCA occlusion	Insular/parietal/peri-ventricular	85
P-05	F	52	14	SAH and R temporal IPH following R MCA aneurysm rupture	Frontal/temporal	11, 12
P-06	M	44	12	R MCA occlusion	Frontal/temporal/parietal	4
P-07	M	72	12	R IPH	Subcortical/basal ganglia	2
P-08	F	46	14	R M1 and ACA occlusion	Basal ganglia	2, 3
P-09	M	55	9	R IPH	Thalamic	2
P-10	F	67	18	R IPH	Frontal/basal ganglia/uncal	150
P-11	M	75	18	R IPH	Frontal/parietal	3
P-12	F	47	12	R MCA occlusion with small hemorrhagic conversion	Frontal/temporal/parietal	4

*R* Right, *MCA* Middle cerebral artery, *SAH* Subarachnoid hemorrhage, *IPH* Intraparenchymal hemorrhage, *ACA* Anterior cerebral artery

Patients were recruited from the inpatient and outpatient rehabilitation facilities of Harborview Medical Center in Seattle, Washington, following referral from treating rehabilitation physicians and therapists based on clinical observation of spatial neglect. They were required to have intact visual fields as determined by confrontation testing, intermediate corrected visual acuity of 20/50 or better, and neglect dyslexia as determined by whole-word or unilateral paralexic errors on the Indented Paragraph Test (Caplan 1987) or reading subtests from the Behavioural Inattention Test (Wilson et al. 1987). See Table 2 for participant scores on these tests. Of note, P-01 did not complete the reading tests as we modified the inclusion criteria after he had enrolled. He did, however, complete the Kessler Foundation Neglect Assessment Process (KF-NAP; Chen et al. 2012), which indicated spatial neglect, and committed neglect dyslexic errors during the experiments described below. Scores from two other patients who completed the KF-NAP as part of their routine clinical care are also detailed in Table 2. Also of note, the traditional scoring methods of the Behavioural Inattention Test and the Indented Paragraph Test do not necessarily reflect error type or lateralization, so we have provided these additional details in Table 2.

**Table 2:** Patient assessment scores

ID	BIT- menu (out of 9 possible points)	BIT- article (out of 9 possible points)	Indented paragraph test	KF-NAP (out of 30 possible points)
P-01	Score: 9 Errors: none	Score: 9 Errors: none	n/a	Score: 8 (mild)
P-04	Score: 9 Errors: none	Score: 9 Errors: 1 (whole-word error on left margin of column 1)	12 errors L: 4% R: 3.5% (1 entire line omission)	n/a
P-05	Score: 7 Errors: 2 (whole-word errors in columns 1 and 3)	Score: 7 Errors: 4 (whole-word errors in columns 1 and 2)	19 errors L: 9.6% R: 3.5% (1 entire line omission)	n/a
P-06	Score: 9 Errors: none	Score: 7 Errors: 6 (5 whole-word errors in all columns; 1 unilateral paralexia)	12 errors L: 2.4% R: 4.5% (3 unilateral paralexias)	n/a
P-07	Score: 9 Errors: none	Score: 1 Errors: 33 (all left marginal whole-word errors except 1; 12 in column 1, 8 in column 2, 13 in column 3)	29 errors L: 12.8% R: 6.5%	n/a
P-08	Score: 1 Errors: 9 (whole-word errors in columns 1 and 3)	Score: 0 Errors: 74 (40 in column 1, 32 in column 2, 2 in column 3; all left marginal whole-word errors and entire lower half omissions of columns 1 and 2)	42 errors L: 21.6% R: 7.5% (2 entire line omissions)	n/a
P-09	Score: 7 Errors: 1 (whole-word error in column 3)	Score: 5 Errors: 14 (10 in column 1, 3 in column 2, 1 in column 3; all left marginal whole-word errors)	174 errors L: 64% R: 47% (13 entire line omissions)	n/a
P-10	Score: 7 Errors: 1 (whole-word errors in column 1)	Score: 5 Errors: 10 (2 in column 1, 3 in column 2, 5 in column 3; 9 left marginal whole-word errors and 1 unilateral paralexia)	47 errors L: 17.6% R: 12.5% (10 left marginal whole-word errors; 3 entire line omissions)	n/a
P-11	Score: 9 Errors: none	Score: 9 Errors: 2 (1 in column 1, 1 in column 3)	5 errors L: 3.2% R: 0.5%	Score: 12.5 (moderate)
P-12	Score: 5 Errors: 3 (1 whole-word errors in column 2, 2 in column 3)	Score: 0 Errors: 77 (53 in column 1, 19 in column 2, 5 in column 3; entire omission of column 1)	56 errors L: 36.8% R: 5% (1 entire line omission)	Score: 10 (mild)

*BIT* Behavioral inattention test (Wilson et al. 1987); Indented paragraph test (Caplan 1987), *KF-NAP* Kessler foundation neglect assessment process (Chen et al. 2012)

Exclusion criteria included evidence of left hemisphere infarct or history of prior neurologic incident, reading disability, intraocular disease, retinopathy, diplopia, aphasia, or alexia. In addition, patients were required to obtain at least 50% correct responses during the staircase procedure with a maximum display duration of 0.2 s, to prevent eye movements from the fixation cross. One enrolled patient was dismissed as a screen failure for this reason. Another patient who had initially shown neglect dyslexia on screening tests was dismissed as a screen failure because his symptoms rapidly resolved in the days between screening and testing.

Seven control participants were recruited by convenience using identical eligibility criteria with the exception of stroke. The control participants were 85.7% female and 14.3% male with a mean age of 44.9 ± 17.0. At the time of testing, three had obtained a PhD, two had a master’s degree, one had a bachelor’s degree, and one had completed high school. They were not matched to the patients by any of these factors because our primary interest was to compare patient performance between Experiments 1 and 2, rather than to compare performance of the patient and control groups, which has been investigated in prior studies.

### Sample size estimation

In the absence of pilot data, we based our sample size on the six participants in Siéroff and Urbanski (2002), described above. Nevertheless, a post hoc analysis of the appropriate sample size can provide some insight into the power of our experiments. The analysis uses the results of Experiment 1 which replicated prior extinction experiments to estimate an appropriate sample size for the more novel Experiment 2. In this analysis, the focus is on the comparison of percent correct for left-sided target words to right-sided target words; and is restricted to the distractor conditions because Experiment 2 measures the left–right difference with a distractor present. In our Experiment 1, the increase in percent errors for the left word compared to the right word was 40.2% with a standard deviation of 13.9%. We based our sample size calculation on a paired t-test with two tails and assumed a power of 80% (alpha error = 0.05). To detect a left-sided effect of 15% (compared to 40% found in Experiment 1), we would need a minimum sample size of 9. With a sample size of 10 in our Experiment 2, this experiment can be expected to detect a 15% difference between errors on the left and right.

### Stimuli and apparatus

Stimuli were drawn randomly from a list of 539 common four-letter English words. Targets and distractors were never identical within a trial and were orthographically and semantically unrelated unless by chance. Words were selected based on having one or more valid orthographic neighbors that could be formed by exchanging the first letter (e.g., CARE, DARE, and FARE) to increase sensitivity to unilateral paralexias (Miceli and Capasso 2001; Behrmann et al. 2002; Ptak et al. 2012; Reinhart et al. 2016).

Stimuli were presented with MATLAB (The MathWorks Inc., Natick, MA, USA) and Psychophysics Toolbox (Brainard 1997). Words were presented at the horizontal meridian of the screen in all capital, white 24-point Courier font with variable luminance against a dark background. Each word subtended a visual angle of 1.6°. A first group of controls (*n* = 4) completed testing in our laboratory. Stimuli were presented in a dimly lit room on a calibrated ViewSonic PF790 monitor with a maximum luminance of 104 cd/m^2^ and an approximate black level of 1.0 cd/m^2^. Participants maintained a 60 cm distance from the monitor with the use of a chinrest.

Patients and a second group of controls completed testing in a secluded room at the hospital, a classroom, or in their home. Stimuli were presented in a dimly lit room on a calibrated HP EliteDisplay E190i monitor with a maximum luminance of 216 cd/m^2^ and an approximate black level of 0.5 cd/m^2^. Participants intermittently held a string secured to the base of the monitor to their nose to maintain a 60 cm distance.

### Procedure

Luminance and display durations were customized for each participant. Blocks of ten trials with a solitary word were presented with the relative luminance and display duration adjusted so that participants obtained 50–80% accuracy. Display duration was kept below 0.2 s to prevent eye movements. To make equivalent the difficulty of parafoveal targets in Experiment 1 and foveal targets in Experiment 2, we adjusted luminance and duration to match performance in solitary conditions only.

**Fig. 1:**
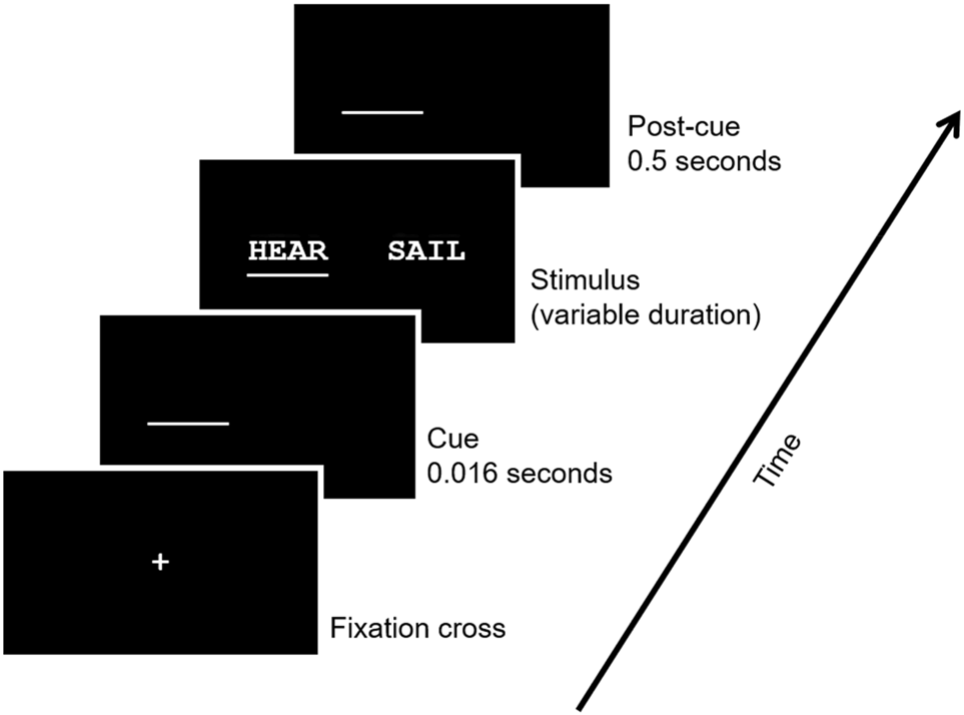
Stimulus sequence. First, subjects fixated on a central cross. After fixation, an underline cue was presented for 0.016 s in the spatial position of the target word. The stimulus was then presented for a duration individualized for each subject with the underline remaining under the target word. Finally, the underline remained as a post-cue for 0.5 s

The sequence of stimulus presentation is shown in Fig. 1. First, the participant fixated on a central cross. Controls used a keypress to advance the stimulus display while most patients used a verbal signal for the experimenter to advance the display due to sensorimotor deficits. Second, the central cross disappeared, and an underline cue was briefly presented at the spatial position of the target word for 0.016 s. Third, the target word appeared above the underline with or without a distractor word for a duration based on the staircase procedure described above. Finally, the underline remained as a post-cue for 0.5 s after the target word was removed. The task was to read aloud or spell out the target word and ignore any distractors. There was no time constraint for the response. The experimenter confirmed or clarified each response by repeating the word back or confirming its spelling.

### Scoring and analysis

After testing was completed, responses were scored as follows. First, responses with more or fewer than four characters were modified to be four characters long and, with an error symbol “X”, reflect the place of the omission or addition error within the word. Modifications maximized the congruence of each letter position between target and response. For example, an addition error such as “CHART” for the target “CART”, would be modified to “XART”; an omission error such “SEE” for the target “SEEM”, would be modified to “SEEX”. Next, each character of the response was compared to each character in the corresponding target and distractor words. Each response was coded to reflect accuracy of each character position. Coded responses were categorized into the following six error types: left contiguous errors (− +  +  + , – +  + , or –− +), right contiguous errors (+ +  + −, +  + –, or + –−), center contiguous errors (+ + − −+ , +—+  + , or + – +), non-contiguous errors (− + − −+ ,− − + –, + − + −, – + −, or − −+  + −), omissions (−−), and intrusions of the distractor word (+ +  +  + for distractor word).

## Experiment 1

Experiment 1 was conducted to ensure our methods induced a similar distractor effect with our patients as found in prior studies. Additionally, we sought to determine if there was a spatial and/or distractor effect on whole-word errors and/or unilateral paralexias.

### Methods

**Fig. 2:**
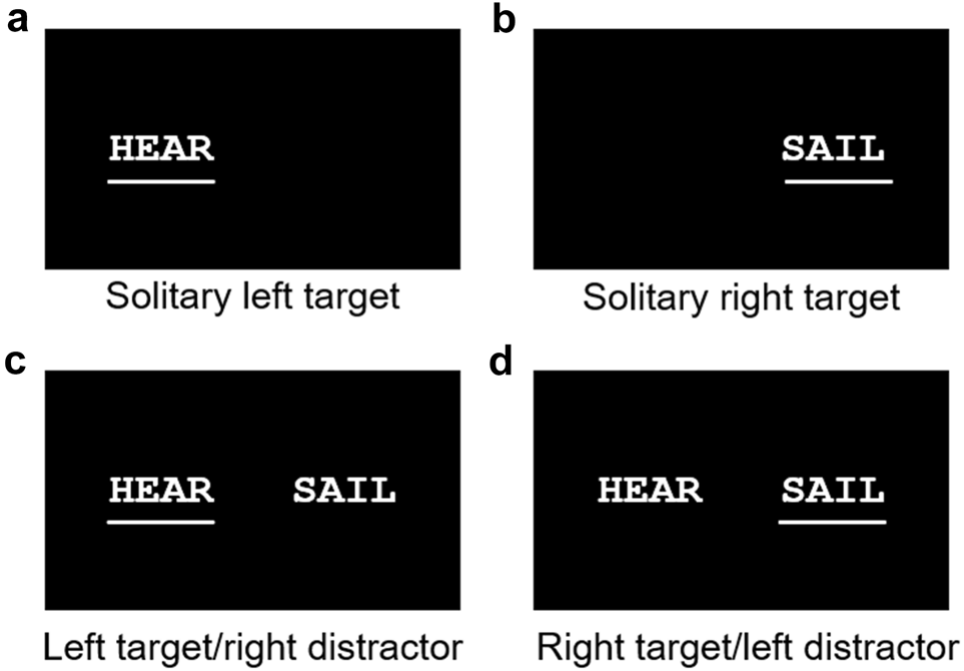
Experiment 1 conditions. The four conditions of Experiment 1 included: **a** a solitary left target condition; **b** a solitary right target condition; **c** a left target/right distractor condition; and **d** a right target/left distractor condition

The four conditions used in Experiment 1 are shown in Fig. 2. In two conditions, the target word was presented solitarily either to the left or right. In the other two conditions, a contralateral distractor word was presented simultaneously. For all patients and two controls, targets and distractors were positioned with their midpoint 1.5° to the left or the right of the fixation cross. Four of the early controls were tested on a different apparatus with 3° spacing and our first control tested on the primary apparatus with 2° spacing as we made minor adjustments to the details of the experiment. Conditions were randomized in blocks of 72 trials, with patients completing 3–5 blocks for an average total of 268 trials and controls completing 1–2 blocks for an average total of 134 trials. For patients, 0.1% of trials were discarded due to the occurrence of an eye movement (determined by visual observation), experimenter error, or participants reporting they were not ready.

### Results

Data from six patients and seven controls were included in these analyses. Patients required a mean relative luminance of 87% and a mean duration of 0.18 s while controls required a mean relative luminance of 34% and a mean duration of 0.11 s. In the right solitary condition, easiest for both groups, performance was roughly matched (34.7 ± 8.6% for patients versus 23.8 ± 4.0% total errors for controls). Thus, patients needed substantially higher luminance and longer display durations to achieve comparable accuracy to controls on their ipsilesional right side.

#### Total errors

To establish that we replicated the main findings of Siéroff & Urbanski (2002), we briefly report total errors. Patients committed a significant 35.4 ± 5.6% more total errors for left-sided than right-sided targets (*F*(1,5) = 40.6, *p* = 0.001, 95% CI[21.1%, 50.0%], Cohen’s *d* = 2.6). The effect of adding a distractor was a significant 14.6 ± 2.0% increase in total errors (*F*(1,5) = 57.2, *p* < 0.001, 95% CI[9.6%,19.6%], Cohen’s *d* = 3.1).

#### Whole-word errors

**Fig. 3:**
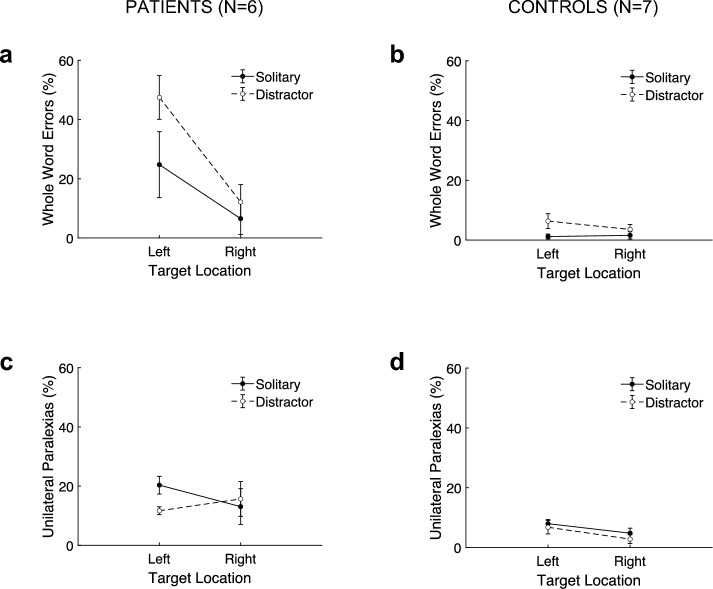
Experiment 1, whole-word errors and unilateral paralexias. Percentages of whole-word errors (top panels) and unilateral paralexias (bottom panels) are plotted as a function of target location. The filled symbols joined by a solid line are the two solitary conditions and the open symbols joined by a dashed line are the distractor conditions

Whole-word errors are plotted in panels A and B of Fig. 3. Patients committed a significant 26.8 ± 8.0% more whole-word errors for left-sided than right-sided targets (*F*(1,5) = 11.4, *p* = 0.020, 95% CI[6.3%,47.2%], Cohen’s *d* = 1.4). The effect of adding a distractor was a significant 14.2 ± 2.7% increase in whole-word errors (*F*(1,5) = 28.0, *p* = 0.003, 95% CI[7.3%,21.0%], Cohen’s *d* = 2.2). Controls committed a non-significant 1.2 ± 1.5% more whole-word errors for left-sided than right-sided targets (*F*(1,6) = 0.6, *p* = 0.466, 95% CI[-2.5%,4.9%], Cohen’s *d* = 0.3). For controls, the effect of adding a distractor was a significant 3.6 ± 1.2% increase in whole-word errors (*F*(1,6) = 9.4, *p* = 0.022, 95% CI[0.7%,6.4%], Cohen’s *d* = 1.2).

##### Intrusions

Many responses in distractor conditions exactly matched the distractor word, a type of whole-word error we call *intrusions*. For patients, intrusions occurred in 16.4 ± 6.2% of trials with left-sided targets, which was significantly different than zero (*t*(5) = 2.6, *p* = 0.02, one-tailed, Cohen’s *d* = 1.1); and in 2.9 ± 1.1% of trials for right-sided targets, which was also significantly different than zero (*t*(5) = 2.7, *p* = 0.02, one-tailed, Cohen’s *d* = 1.1). Patients committed a significant 13.5 ± 5.4% more intrusions for left-sided than right-sided targets (*t*(5) = 3.6, *p* = 0.016, 95% CI[3.8%,23.2%], Cohen’s *d* = 1.2). With left-sided targets, distractors increased errors by 19.5 ± 3.6%. Of these errors 16.4 ± 6.2% were intrusions. Thus, they were the bulk of the additional errors caused by the distractor.

Controls committed few intrusions for left-sided targets (0.8%), which were not significantly different than zero (*t*(6) = 1.6, *p* = 0.086, one-tailed, Cohen’s *d* = 0.7), and no intrusions for right-sided targets. They had a non-significant 0.8 ± 0.5% more intrusions for left-sided than right-sided targets (*t*(6) = 2.2, *p* = 0.071, 95% CI[0,1.6%], Cohen’s *d* = 0.8).

#### Unilateral paralexias

Unilateral paralexias are plotted in panels C and D of Fig. 3. For patients, these errors occurred in 15.2 ± 2.2% of trials across all conditions and were significantly more common than right-sided contiguous errors (i.e., reversed unilateral paralexias), which occurred in 5.6 ± 1.0% of trials (*t*(23) = 3.5, *p* = 0.002, 95% CI[3.9%,15.3%], Cohen’s *d* = 1.6). Patients committed a non-significant 1.6 ± 6.4% more unilateral paralexias for left-sided than right-sided targets (*F*(1,5) = 0.07, *p* = 0.808, 95% CI[−14.7%,18.0%], Cohen’s *d* = 0.1). In distractor conditions, patients had a significant 3.0 ± 1.0% fewer unilateral paralexias (*F*(1,5) = 9.1, *p* = 0.030, 95% CI[−5.6%,−0.4%], Cohen’s *d* = -1.3).

Controls did commit unilateral paralexias but their occurrence, 5.6 ± 0.9% of trials across all conditions, was not significantly greater than right-sided contiguous errors, which occurred in 7.7 ± 1.3% of trials (*t*(27) = −1.4, *p* = 0.161, 95% CI[−5.3%,0.9%], Cohen’s *d* = −0.7). Controls committed a non-significant 3.6 ± 1.8% more unilateral paralexias for left-sided than right-sided targets (*F*(1,6) = 3.9, *p* = 0.097, 95% CI[−0.9%,8.0%], Cohen’s *d* = 0.7). In distractor conditions, controls had a non-significant 1.6 ± 1.6% fewer unilateral paralexias (*F*(1,6) = 1.0, *p* = 0.348, 95% CI[−5.4%,2.2%], Cohen’s *d* = −0.4). In summary, there was little effect of side and a small, reversed effect of distractors, especially for the left side. Controls had relatively few unilateral paralexias that were no more common than right-sided contiguous errors and they did not significantly vary with side or distractor.

### Discussion

In Experiment 1, patients had more whole-word errors for left-sided targets and in distractor conditions. Intrusions comprised a substantial portion of whole-word errors in distractor conditions, particularly on the left side. Unilateral paralexias accounted for nearly a third of errors. The reduction in unilateral paralexias in distractor conditions can be explained by the increase in whole-word errors in those conditions (a whole-word error would make the word unavailable for other error processes). Besides this effect, unilateral paralexias were not modulated by spatial position or the presence of a distractor. In summary, there were spatial and distractor effects for whole-word errors but not for unilateral paralexias.

## Experiment 2

The traditional extinction paradigm of Experiment 1 is unable to discern whether errors in the left target/right distractor condition are attributable to their position relative to the viewer or to their position in a two-word allocentric frame. Therefore, the purpose of Experiment 2 is to determine whether the distractor effect observed with parafoveal targets and distractors occurs with foveal targets and parafoveal distractors.

### Methods

**Fig. 4:**
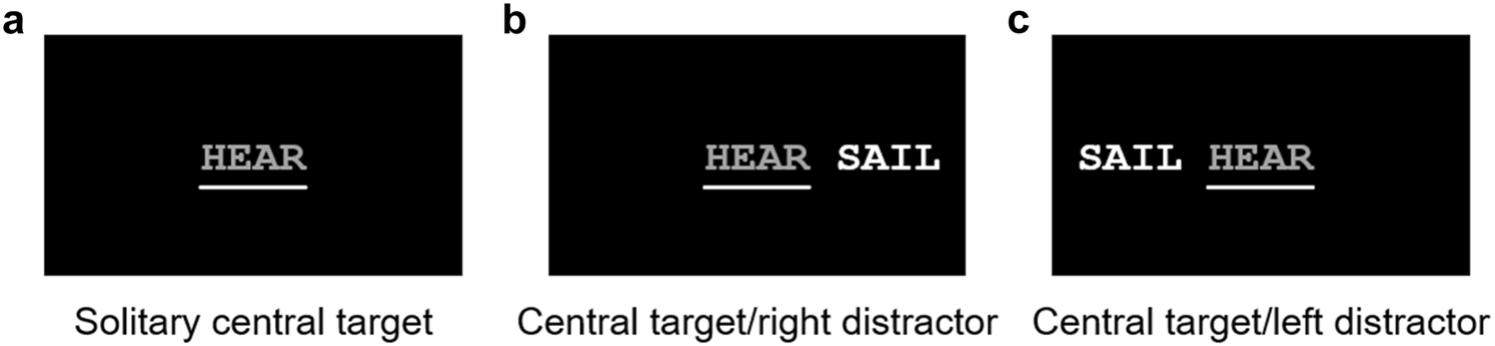
Experiment 2 conditions. The three conditions of Experiment 2 included: **a** a solitary central target condition; **b** a central target/right distractor condition; and **c** a central target/left distractor condition. Note that the distractor word has an increased relative luminance compared to the target word

Figure 4 shows the three conditions used in Experiment 2: a solitary central condition, in which the target word was presented foveally; and two distractor conditions, in which a distractor word was presented to the left or right side of the foveal target word. Distractor words were positioned with their midpoint 2.5° from center for all patients and two controls. Distractor words for three early controls were positioned with their midpoint 3° from center before minor adjustments to the details of the experiment. Trials were randomized in blocks of 72 trials, with controls completing 1–2 blocks for an average of 134 trials and patients completing 3–8 blocks for an average of 318 trials. For patients, 0.4% of trials were discarded due to the occurrence of an eye movement, experimenter error, or participants reporting they were not ready.

As in Experiment 1, we used a modified staircase procedure to customize the relative luminance and display durations for each participant to obtain a desired performance level. Distractors were presented at a relative luminance of 3 × that of targets to equalize their visibility, based on a pilot experiment completed with our first group of controls.

### Results

Patients had a mean relative luminance of 32% and a mean display duration of 0.08 s, resulting in 39.5 ± 3.3% total errors in the solitary condition. Controls had a mean relative luminance of 2% and a mean display duration of 0.03 s, resulting in 38.1 ± 6.0% total errors in the solitary condition.

#### Whole-word errors

**Fig. 5:**
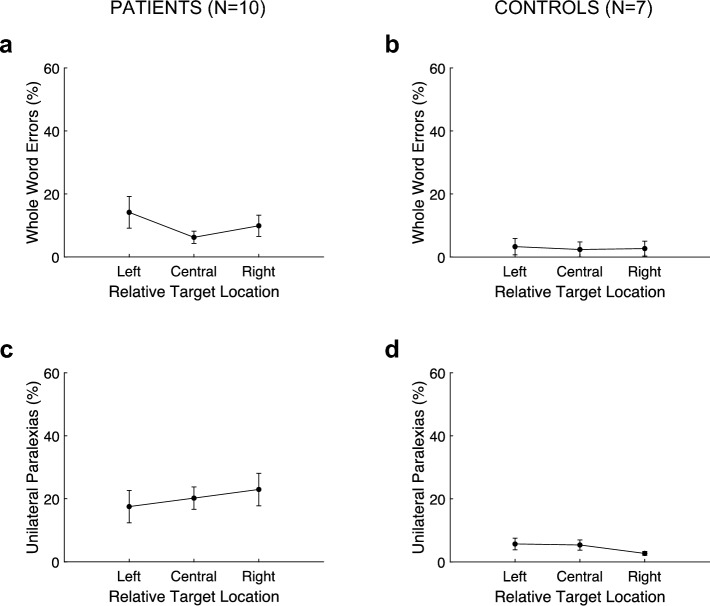
Experiment 2, whole-word errors and unilateral paralexias. Percentages of whole-word errors (top panels) and unilateral paralexias (bottom panels) are plotted as a function of relative target location. Thus, the right distractor condition is plotted on the left and the left distractor condition is plotted on the right

Whole-word errors are plotted in panels A and B of Fig. 5. Patients committed a significant 5.8 ± 2.1% more whole-word errors in distractor conditions than in the solitary condition (*F*(1,9) = 7.3, *p* = 0.014, 95% CI[1.3%,10.3%], Cohen’s *d* = 0.9). There was a non-significant effect of distractor side, with 4.3 ± 2.5% more errors with right-sided than left-sided distractors (*F*(1,9) = 3.0, *p* = 0.102, 95% CI[−9.5%,0.9%], Cohen’s *d* = −0.6). Intrusions occurred on 2.6 ± 1.2% of targets with right-sided distractors, which was significantly different than zero (*t*(9) = 2.2, *p* = 0.026, one-tailed, Cohen’s *d* = 0.7); but on only 0.2 ± 0.2% of targets with left-sided distractors, which was not significantly different than zero (*t*(9) = 1.0, *p* = 0.172, one-tailed, Cohen’s *d* = 0.3). A significant 2.4 ± 1.0% more intrusions were committed with right-sided than left-sided distractors (*t*(9) = 2.0, *p* = 0.022, 95% CI[−0.3%,5.1%], Cohen’s *d* = 0.9). In summary, patients had more whole-word errors in the distractor conditions than the solitary condition, but without a significant difference for distractor side. Intrusions occurred more often when the target word was in the relative left position.

Controls committed a non-significant 0.6 ± 0.3% more whole-word errors in distractor conditions than in the solitary condition (*F*(1,6) = 3.4, *p* = 0.09, 95% CI[−0.1%,1.3%], Cohen’s *d* = 0.7) and a non-significant 0.6 ± 0.4% more whole-word errors with right-sided distractors than with left-sided (*F*(1,6) = 2.6, *p* = 0.135, 95% CI[−1.4%,0.2%], Cohen’s *d* = −0.6). Controls committed no intrusions in Experiment 2.

#### Unilateral paralexias

Unilateral paralexias are plotted in panels C and D of Fig. 5. For patients, unilateral paralexias were committed in 20.2 ± 2.6% of trials across all conditions and were significantly more common than right-sided contiguous errors, which occurred in 4.3 ± 0.6% of trials (*t*(29) = 5.4, *p* < 0.001, 95% CI[9.9%,22.0%], Cohen’s *d* = 1.7). Patients committed a non-significant 0.02 ± 3.0% more unilateral paralexias in distractor conditions than in the solitary condition (*F*(1,9) < 0.1, *p* = 0.996, 95% CI[−6.4%,6.4%], Cohen’s *d* = 0.002) and a non-significant 5.4 ± 3.5% more with left-sided distractors than with right-sided (*F*(1,9) = 2.4, *p* = 0.139, 95% CI [−1.9%,12.8%], Cohen’s *d* = 0.5). In summary, for patients, unilateral paralexias comprised a substantial portion of total errors and remained consistent across all conditions.

Controls committed unilateral paralexias in 4.6 ± 0.9% of trials across all conditions, which were not significantly more common than right-sided contiguous errors, which occurred in 5.5 ± 1.1% of trials (*t*(20) = −0.7, *p* = 0.494, 95% CI[−3.6%,1.8%], Cohen’s *d* = −0.3). Controls committed a non-significant 1.2 ± 1.3% more left-sided errors in the solitary condition than in the distractor conditions (*F*(1,6) = 0.9, *p* = 0.370, 95% CI[−3.9%,1.6%], Cohen’s *d* = −0.4). They committed a non-significant 3.0 ± 1.5% more left-sided errors with right-sided distractors than left-sided (*F*(1,6) = 4.2, *p* = 0.063, 95% CI[−6.2%,0.2%], Cohen’s *d* = −0.8).

### Discussion

In Experiment 2, we modified the traditional extinction paradigm so that the target word was always presented foveally. Whole-word errors were committed more frequently with distractors but were not differently affected by left- or right-sided distractors. Intrusions were more frequent with right-sided distractors but were substantially reduced in comparison to Experiment 1. Also consistent with Experiment 1, unilateral paralexias were common and consistent across conditions.

### Comparison of experiments 1 and 2

Experiments 1 and 2 can be compared to distinguish whether the spatial effects are due to the target’s egocentric position or its position within a two-word allocentric frame. If its egocentric position is critical, then spatial effects found with Experiment 1 should be eliminated in Experiment 2 which has the target word centered in the fovea. Alternatively, if its allocentric position is critical, then both experiments should have similar spatial effects. For whole-word errors, there were large spatial effects in Experiment 1 and near zero effects in Experiment 2. The difference was 31 ± 8% which was statistically significant by a two-sample t-test with unequal variance (*t*(5.57) = 3.98, *p* = . 008, 95% CI[12, 51%], Cohen’s *d* = 1.6). This is consistent with an egocentric frame of reference and not a two-word allocentric frame.

For unilateral paralexias, there were no detectable spatial effects for either experiment. The difference was 1.7 ± 4.5% which was not statistically significant by a two-sample *t* test with unequal variance (*t*(13.6) = 0.38, *p* = 0.709, 95% CI[−8,12%], Cohen’s *d* = 0.1). This is consistent with one-word allocentric frame of reference. Furthermore, using the confidence intervals one can rule out an egocentric effect similar to what was found with whole-word errors. Thus, there are sharply contrasting effects for the two kinds of errors, consistent with different frames of reference.

## General discussion

In this study, we investigated the influence of space and distractors on whole-word errors and unilateral paralexias. Experiment 1 was a replication of the traditional extinction paradigm for words with parafoveal targets and distractors. Experiment 2 used a modified paradigm with foveal targets and parafoveal distractors. Thus, Experiment 2 controlled the effects of egocentric space on targets while manipulating allocentric space with the placement of distractors. In addition, to match visibility with parafoveal and foveal targets, we adjusted the luminance and durations to equate performance for solitary targets.

Regarding whole-word errors, there were two main results. First, by comparing Experiments 1 and 2 we found whole-word errors to depend on the target's egocentric position and not on its position within a two-word allocentric frame. Second, in the presence of a distractor, these whole-word errors were largely made up of intrusions from the distractor word.

Regarding unilateral paralexias, there were two main results. Unilateral paralexias were unaffected by the target word’s egocentric position or its position within a two-word allocentric frame. Second, unilateral paralexias were unaffected by the presence of a distractor word. Both of these effects were in sharp contrast to the effects found for whole-word errors.

### Whole-word errors depend on egocentric space

In the solitary conditions of Experiments 1 and 2, we found a substantial effect of the target word’s egocentric spatial position on whole-word errors. For patients, accuracy improved incrementally from left to right. This gradient in accuracy has been previously reported in several studies of neglect dyslexia (Siéroff and Michel 1987; Miceli and Capasso 2001; Behrmann et al. 2002; Ptak et al. 2012; Moore and Demeyere 2023) and is consistent with an egocentric spatial explanation of whole-word errors. That is, the further a target word is positioned in the left egocentric hemispace, the greater the likelihood that a target word is omitted. Mechanisms that have been proposed to underlie the horizontal gradient include that left-sided sensory input is degraded as compared to right-sided (Bender 1952; Heilman et al. 1985; Farah et al. 1991) or that patients experience an anisometric perception of egocentric space, making the left side appear “relaxed” and the right side “constricted” (Bisiach et al. 1998, 1999).

Experiment 1 showed a spatially biased distractor effect for left-sided targets and right-sided distractors, which effectively replicated Siéroff and Urbanski (2002). However, it could not distinguish whether the effect was dependent on the target’s egocentric position or its position within a two-word allocentric frame. To make this distinction was one of our primary motivations for Experiment 2.

In contrast to the substantial distractor effect for left-sided words in Experiment 1, we found a significant but *non-lateralized* distractor effect for foveal targets with parafoveal distractors in Experiment 2. That is, accuracy of reporting the central target word was equivalently affected by the presence of a distractor on either the left or right side. This is in agreement with several prior studies with non-word stimuli that have shown parafoveal stimuli to have equivalent priming effects (e.g., de Haan et al. 2015; Fuentes and Humphreys 1996; Làdavas et al. 1993) and interference effects (e.g., Audet et al. 1991; Cohen et al. 1995; Diedrichsen et al. 2000; Lavie and Robertson 2001; Ro et al. 1998; Snow and Mattingley 2008) for the detection or identification of a foveal target. However, we did find that intrusions were significant only for right-sided distractors in Experiment 2. Taken together, these results suggest that left-sided stimuli undergo a degree of processing sufficient to interfere with a foveal target, but insufficient to be selected over a foveal target; while right-sided stimuli cause foveal interference and are susceptible to erroneous selection over a foveal target.

These distractor effects can be interpreted as a failure of selective attention, building on the biased competition model, in which multiple stimuli compete for limited processing, mediated by top–down behavioral goals and bottom-up stimulus characteristics (Desimone and Duncan 1995). This lateralized biased competition has been hypothesized to result from a disruption to the reciprocal inhibition of the two cerebral hemispheres (Kinsbourne 1977). Consequent to damage to the neurons in the right hemisphere that encode left-sided spatial locations, an unopposed left hemisphere selects right-sided stimuli for processing at the expense of left-sided stimuli. An alternative account of visual crowding is dismissed because of the wide separation of the words in the distractor conditions of both experiments.

A novel result of our study was frequent intrusions of the distractor word. In fact, in Experiment 1, the bulk of the errors due to distractors were intrusions. They occurred for both left-sided and right-sided targets, but were much more common for left-sided targets. In Experiment 2, however, intrusions were significant only when the target was on the relative left side of the distractor. Similar to intrusions, contralateral mislocalizations termed *dyschiria* or *allochiria* (Meador et al. 1991) have been reported previously in participants with unilateral brain damage—most commonly reported in the tactile modality (e.g., Bisiach and Berti 1987; Kawamura et al. 1987; Meador et al. 1991; Ricci et al. 2019), but also for visuomotor tasks such as copying (Halligan et al. 1992; Lepore et al. 2004) and drawing from memory (Grossi et al. 2004).

There are several possible interpretations of intrusions. One is that the cue was sometimes mislocalized to the opposite position within the two-word pair. If a left-sided cue (or relative left-sided cue in Experiment 2) was mislocalized to the right hemifield, it is natural that the right-sided distractor word was reported rather than the target word. An alternate explanation is that the cue was more-or-less correctly localized, but the left-sided word was poorly localized. In that case, the right-sided distractor might have been judged as closest to the cued location. Such selection errors occur in normal vision when the cues and competing stimuli are close together in the periphery (Palmer and Moore 2009; Yiǧit-Elliott et al. 2011).

Alternatively, intrusions might not signify any deficit beyond one of selective attention. For example, patients might have preferred to provide an incorrect response (i.e., the distractor word) rather than a vacant response (i.e., “I don’t know”). Regardless of the mechanism underlying these intrusion errors, they are supportive of some kind of deficit in selective attention.

### Unilateral paralexias remain consistent across egocentric space

We found a relatively consistent pattern of unilateral paralexias throughout both experiments. The reduction of unilateral paralexias in the distractor conditions can be explained by the reciprocal increase in whole-word errors: such a complete failure of word recognition removed the opportunity for unilateral paralexias. The consistency of unilateral paralexias suggests a distinct within-word allocentric deficit that is not modulated by spatial position or the presence of a distractor.

Ptak et al. (2012) reported a similarly consistent rate of unilateral paralexias across egocentric space when they presented 40 words distributed in five columns on a single sheet of paper. Similarly, Miceli and Capasso (2001) reported, for a single participant, comparable error rates for the first letter position of 4-letter words when presented centrally or to the left or right of fixation. The error rate for the first letter position of right-sided targets, for example, was 31.7%, while the error rate for the third letter of the centrally presented word, which occupied the identical egocentric position, was just 2.7%.

There is evidence that single words are perceived in a similar manner to objects. First, substitution errors (e.g., NEAR→BEAR) are more common than omission errors (e.g., NEAR→EAR) or addition errors (e.g., NEAR→CLEAR; Arduino et al. 2002a, b; Arguin and Bub 1997; Behrmann et al. 1990). Second, words are read more accurately than non-word strings (Siéroff et al. 1988; Behrmann et al. 1990; Arduino et al. 2002b), unless the cohesion of a word is manipulated through increasing the spacing between letters (Siéroff 1991). Third, unilateral paralexias have been reported to occur for words positioned in the right egocentric hemispace (Kinsbourne and Warrington 1962; Siéroff 1991; Ptak et al. 2012).

Further evidence for a distinct within-word allocentric spatial impairment is provided by our result of no significant effect of the presence of a distractor on unilateral paralexias in either experiment. Furthermore, we found no significant difference in unilateral paralexias in conditions with left- versus right-sided distractors.

## Conclusion

In this study, we manipulated the spatial position of target and distractor words within egocentric and allocentric frames to determine the spatial and attentional contributions to whole-word errors and unilateral paralexias in patients with neglect dyslexia. For whole-word errors, we found both spatial and distractor effects with parafoveal targets and distractors, but only distractor effects with foveal targets and parafoveal distractors. In addition, we found the errors with distractors to be largely intrusions of the distractor word. This reinforces previous interpretations of this extinction effect as a failure of selective attention. In contrast, we found unilateral paralexias to remain largely consistent throughout manipulations of spatial position and the presence of a distractor. Thus, these errors are primarily a function of allocentric space within a word with no sign of an effect of egocentric space. In summary, our findings support an account of neglect dyslexia based on three distinct deficits: an egocentric deficit across space resulting in whole-word errors, a failure of selective attention resulting in whole-word intrusion errors, and an allocentric deficit within a word resulting in unilateral paralexias.

## Data Availability

The dataset generated from this study is available at: https://osf.io/7czse.
